# miR-151-5p, targeting chromatin remodeler SMARCA5, as a marker for the BRCAness phenotype

**DOI:** 10.18632/oncotarget.10345

**Published:** 2016-06-30

**Authors:** Stefania Tommasi, Rosamaria Pinto, Katia Danza, Brunella Pilato, Orazio Palumbo, Lucia Micale, Simona De Summa

**Affiliations:** ^1^ IRCCS Istituto Tumori “Giovanni Paolo II”, Molecular Genetics Laboratory, Bari, Italy; ^2^ IRCCS ‘Casa Sollievo della Sofferenza’, Medical Genetics Unit, San Giovanni Rotondo (FG), Italy

**Keywords:** breast cancer, BRCAness, microRNA profiling, DNA repair, miR-151-5p

## Abstract

In recent years, the assessment of biomarkers useful for “precision medicine” has been a hot topic in research. The involvement of microRNAs in the pathogenesis of breast cancer has been highly investigated with the aim of being able to molecularly stratify this highly heterogeneous disease. Our aim was to identify microRNAs targeting DNA repair machinery, through Affymetrix GeneChip miRNA Arrays, in a cohort of BRCA-related and sporadic breast cancers. Moreover, we analyzed microRNA expression taking into account our previous results on the expression of PARP1, because of its importance in targeted therapy. miR-361-5p and miR-151-5p were found to be overexpressed in PARP1-upregulating BRCA-germline mutated and sporadic breast tumors. Pathway enrichment analysis was performed to identify potential target genes to be analyzed in the validation step in an independent cohort. Our results confirmed the overexpression of miR-151-5p and, interestingly, its role in the targeting of SMARCA5, a chromatin remodeler. This result was also confirmed *in vitro*, both through luciferase assay and by analyzing endogenous levels of SMARCA5 in MCF-7 cell lines using miR-151-5p mimic and inhibitor. In conclusion, our data showed the possibility of considering the overexpression of PARP1 and miR-151-5p as biomarkers useful to correctly treat sporadic breast cancers, which eventually could be considered as BRCAness tumors, with PARP-inhibitors.

## INTRODUCTION

BRCA1 and BRCA2 genes are considered susceptibility genes in hereditary breast cancer but are also involved in important metabolic functions of cell life. They function as tumor suppressors and are important in the maintenance of genomic stability through their role in DNA damage signalling and DNA repair. Both of them are implicated in mediating repair of double strand breaks (DSBs) *via* homologous recombination (HR) through interactions with RAD51. Cells deficient in BRCA1/2 are unable to repair DSBs by error-free HR, resulting in repair by the error-prone non-homologous end joining (NHEJ) pathway introducing chromosomal instability [1, 2]. As inactivation of BRCA1/2 leads to impaired HR, it has been investigated whether mutation carriers would be sensitive to DNA cross-linking agents, such as platinum salts, as they introduce DSBs. Indeed, high response rates to cisplatin have been demonstrated in BRCA1 mutation carriers [3, 4]. Recently, a novel targeted therapy, PARP inhibitors (PARPi), based on a “synthetic lethality” approach [5], has emerged in patients carrying germline BRCA1/2 mutations. Briefly, PARP1 is a player of the base excision repair pathway, which is responsible for removing damaged bases by mechanisms such as deamination, oxidation, and alkylation. PARPi lead to accumulation of single strand breaks which, if unrepaired, become DSBs which are not processable in BRCA-deficient cells. Early clinical trials demonstrated a significant efficiency of PARPi in BRCA-deficient breast and ovarian cancers [6-8]. Because of the phenotypic similarities between BRCA1-related and triple negative BCs (TNBCs), a phase 2 study was conducted to test the efficiency of iniparib, in addition to standard chemotherapy, in metastatic TNBCs, with promising results [9]. However, the phase 3 clinical trial failed to show significant improvements, probably due to a lack of assessment of BRCA1/2 mutational status. There is therefore an emerging need to select patients to direct to such a treatment, even in the absence of BRCA1 or BRCA2 pathological mutations [10]. However, a comprehensive view of DDR machinery needs to be considered through the “access-repair-restore” model. Such a model, proposed in 1991, explained that, after identification of DNA damage, chromatin structures undergo modifications that make them accessible for repairing and then return to their initial status [11]. Thus, genes involved in the regulation of the accessibility to chromatin will also be considered in the present study.

The primary aim of our paper is to identify specific miRNAs involved in a phenotype druggable by PARPi. About 10-20% of TNBCs are estimated to be deficient in BRCA1/2 and additional cases are thought to have BRCA1/2 deregulation due to epigenetic effects, e.g. BRCA1-promoter methylation or microRNAs (miRNAs) which target BRCA1/2 genes, decreasing their levels.

Larsen et al [12] showed that genome-wide RNA profiling and BRCA1 promoter methylation analyses could be able to characterize familial non-BRCA1/2 tumors and to discriminate BRCA1-like BCs displaying the so-called “BRCAness” phenotype, intending with this term tumors with defects on repairing DSBs. We reviewed [13] how such a phenotype could not simply be addressed to TNBCs or basal-like tumors. Moreover, we previously indicated the possibility to evaluate the overexpression of PARP1 and miR-17, which targets BRCA1, as biomarkers of BRCAness BCs [14]. Starting from these results we performed miRNA profiling with the aim to identify deregulated miRNAs targeting DNA repair machinery.

## RESULTS

### miRNA expression profiling in sporadic and BRCA1/2-mutated patients according to PARP1 gene expression

**Figure 1:**
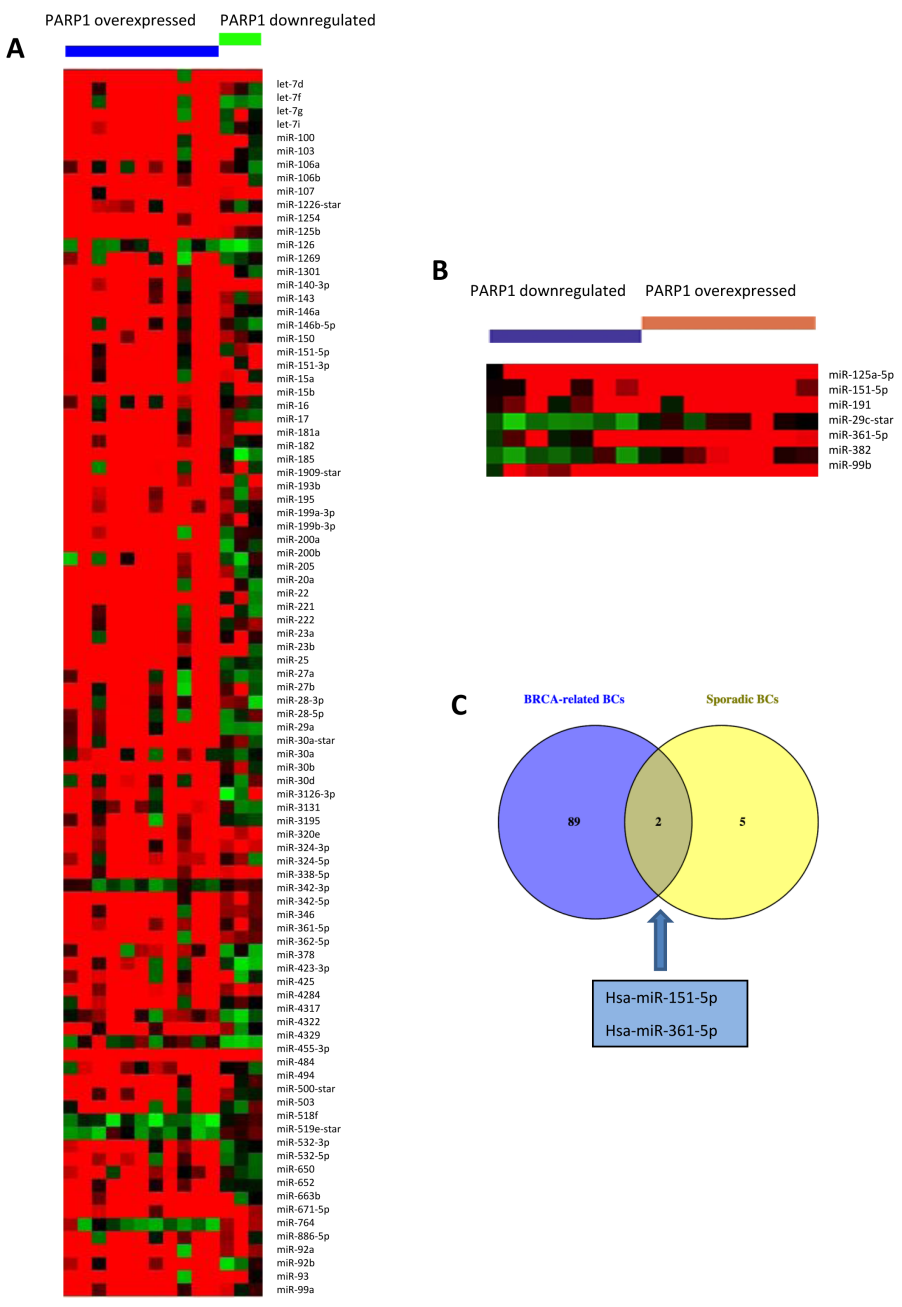
miRNAs deregulated in **A.** BRCA-related and **B.** sporadic BCs, according to PARP1 expression status. Deregulated miRNAs were identified through the *t*-test, considering results as significant when *p* < 0.01. **C.** Venn diagram showing the two miRNAs upregulated in PARP1-overexpressing cases, both mutated and sporadic.

In our previous study [14], we suggested that the upregulation of PARP1 and miR-17, which targets the BRCA1 gene, could be a marker of the BRCAness phenotype in sporadic patients. In order to better explore if other miRNAs could be considered a marker of the BRCAness phenotype, miRNA expression profiling was performed in BRCA1/2 germline mutation positive and sporadic patients, excluding the familial BRCAX subset (BRCAX patients are familial BCs not carrying BRCA-germline mutations). We selected miRNAs annotated as ‘hsa’ in order to exclusively analyze the differential expression of human genes. The selected hsa-miRNAs (*n* = 1100) underwent statistical analysis by *t*-test performed through MeV software. We were interested in the identification of miRNAs deregulated concomitantly to PARP1 overexpression, thus we stratified both mutated and sporadic samples according to PARP1 expression. We identified 94 deregulated miRNAs in BRCA-related cases and 7 in sporadic BCs (Figure 1A-1B). As shown in Figure 1C, we found that miR-151-5p and miR-361-5p were both upregulated in PARP1-overexpressing BRCA-mutated (*n* = 11) and sporadic BCs (*n* = 8) when compared to PARP1-downregulating cases.

### Bioinformatic evaluation of miRNA targets

**Figure 2:**
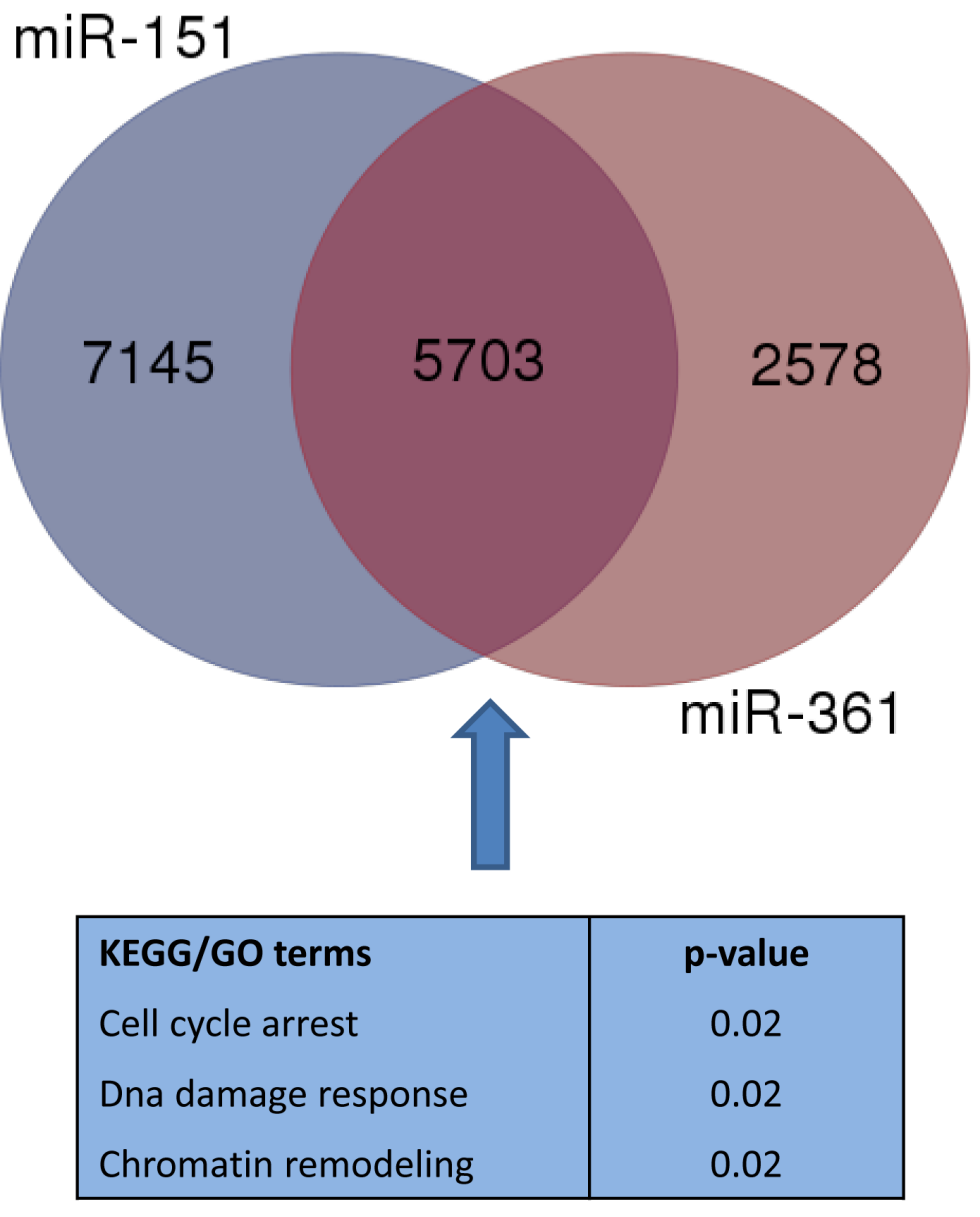
Venn diagram displaying the overlap of enriched KEGG/GO terms considering predicted targets of miR-151-5p and miR-361-5p We highlighted terms which were considered to identify target genes which underwent validation.

Bioinformatic analyses were performed in order to identify gene targets to be included in the validation step. The miRwalk database was queried for predicted targets of miR-151-5p and miR-361-5p. In Figure 2, it could be observed that 5703 predicted targets were shared by the two miRNAs, and they underwent pathway enrichment analysis through DAVID, including GO_BP and KEGG terms. We focused on terms related to “DNA damage response”, “cell cycle arrest” and “chromatin remodeling”. After literature checking, we decided to include in the validation step ATM, WEE1 and SMARCA5 genes as potential targets of miR-151-5p and miR-361-5p.

### Validation of miRNAs and targeted genes in an independent cohort

**Figure 3:**
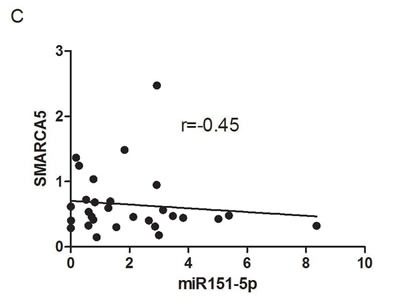
Results in the validation cohort Relative expression of miR-151-5p in **A.** BRCA-related and **B.** sporadic BCs. **C.** Correlation between miR-151-5p and SMARCA5 relative expressions.

We performed the validation step in an independent cohort including 8 BRCA-related and 21 sporadic BCs. They were stratified according to gene expression level of PARP1, considering the median value as cut-off to define samples up/down-regulating it. 50% of BRCA-related and 52.3% of sporadic BCs showed upregulation of PARP1. Only miR-151-5p expression resulted validated, while miR-361-5p was not found to be overexpressed in PARP1-upregulated BCs, either BRCA-related or sporadic, and was then excluded from the subsequent analyses. The median expression value of miR-151-5p is lower both in PARP1- overexpressing BRCA-related and sporadic BCs as shown in Figure 3A-3B. The dispersion of continuous data in such a small sample set could not reach statistical significance. For this reason, we performed two-tailed Fischer's exact test to verify the frequencies of miR-151-5p overexpressing patients. We found that 100% of BRCA-related and 81.8% of sporadic PARP1-overexpressing BCs overexpressed miR-151-5p (*p* = 0.01). Moreover, looking at the expression of its potential gene targets, an inverse correlation between SMARCA5 and miR-151-5p expressions was found (Figure 3C) in the entire validation set (*r* = -0.45). Regarding ATM and WEE1, we did not find any inverse correlation between miR-151-5p and their expression (data not shown). As the inverse relationship between miR-151-5p and its target SMARCA5 did not reach statistical significance due to the small sample size, a functional study to verify their interaction was performed.

### Functional analysis to test SMARCA5 regulation miR-151-5p

To determine whether *SMARCA5* is really targeted by miR-151-5p, we first screened the sequence of the human *SMARCA5* gene by using bioinformatic tools to search for putative miRNA binding sites [15]. As a result, we found a miR-151-5p targeting site in the coding sequence of *SMARCA5*.

**Figure 4:**
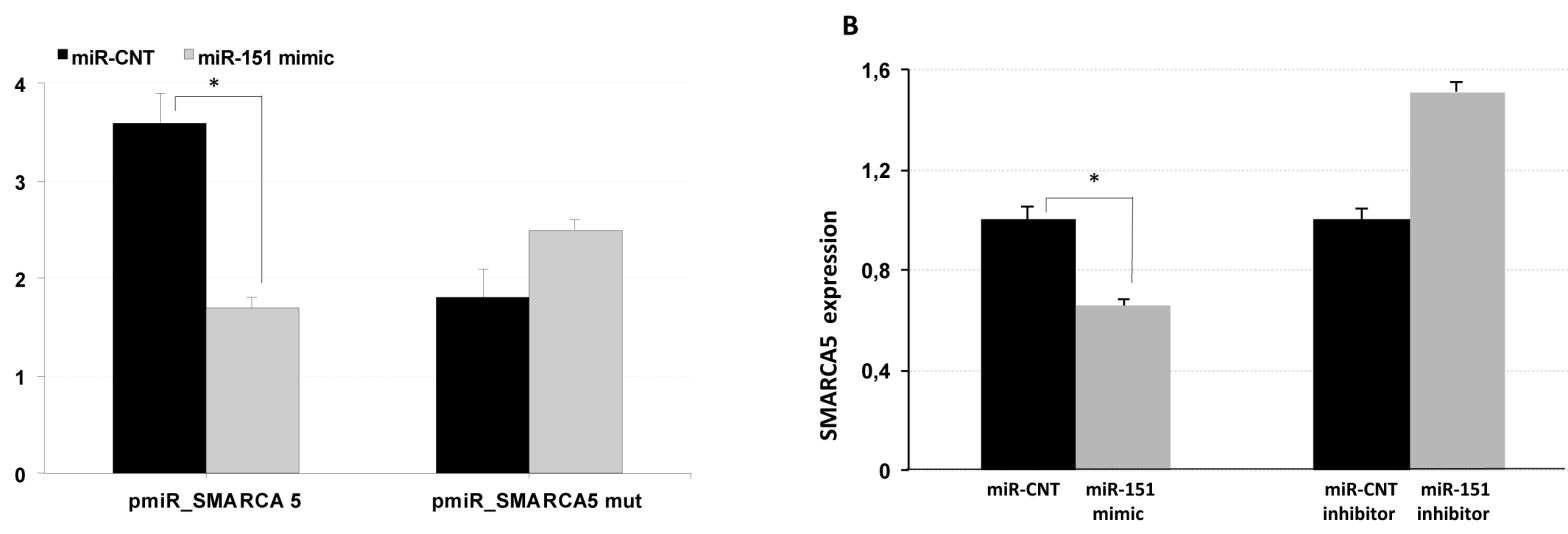
SMARCA is a miR-151 target **A.** HEK293 cells were co-transfected with reporter constructs carrying the encoding sequence of SMARCA containing wild type or mutated miR-151 complementary site and a synthetic mimic of miR-151 or miR-control (miR-CNT). Luciferase activities were analyzed at 48 h post transfection and normalized to the level of the control Renilla luciferase. **B.** Detection of SMARCA endogenous expression by qPCR in MCF-7 cell lines transfected with miR-151 mimic or miR-control or with miR-151 inhibitor or miR-control inhibitor. *p < 0.005.

To experimentally test *in vitro* whether *SMARCA5* is directly targeted by miR-151-5p, we cloned the putative miR-151-5p binding region downstream to a luciferase reporter gene in the pmiR-REPORT Vector. Next, we co-transfected HEK293 cells with the *SMARCA5* reporter construct or a control vector containing a scrambled sequence along with a synthetic mimic of miR-151-5p. We detected that the overexpression of miR-151-5p reduced the luciferase activity of the vector containing the coding sequence of *SMARCA5* when compared to the control, while deletion of the miR-151-5p binding site abrogated this effect (Figure 4A).

Furthermore, we explored whether this regulation occurred for endogenous *SMARCA5*. In keeping with our luciferase data, qPCR results showed a negative correlation between miR-151-5p and *SMARCA5* expression levels in MCF-7 breast cancer lines, while the use of miR-151-5p inhibitor had a slightly increased expression of *SMARCA5* mRNA (Figure 4B). Overall, these results indicated that *SMARCA5* is targeted by miR-151-5p.

## DISCUSSION

DNA damage response (DDR) includes DNA repair mechanisms, cell cycle arrest in order to allow the cell to repair DNA insults and, if unrepaired, induction of apoptosis or senescence [16]. It is well known that cancer affects genomic stability, as reviewed in [17]. Most anti-cancer therapies target the cell cycle inducing DNA damage and thus cell death, but lesions could be repaired by remaining mechanisms. An intriguing approach is to hit functional mechanisms, leading cancer cells to be totally unable to repair pharmacological damage. PARPi represent an example of such an approach as its effectiveness depends on impairment of HR [18, 19] due to genetic/epigenetic alterations of BRCA1/2 genes. However, recently effectiveness of PARPi in PTEN-deficient tumors has also been demonstrated [20]. Thus other factors involved in DDR could be able to improve PARPi sensitivity. The role of miRNAs, which are involved in many biological processes, has also been explored in DDR [21, 22]. In the present study, we focused on the identification of miRNAs targeting DDR genes overexpressed both in BRCA1/2 germline mutation positive and sporadic BCs. Moreover, given that PARP1 is the therapeutic target, we also considered its expression to stratify our cohort. In our previous study, we showed the co-expression of miR-17 and PARP1 [14] and the same cohort underwent miRNA expression profiling. Our aim was to highlight similarities between these BRCA-related and sporadic BCs in order to be able to better stratify the former and to identify sporadic cases which could be treated with PARPi. Indeed, this therapeutic approach is not effective in all BCs carrying mutations in BRCA1 or BRCA2 genes [23] and it is also known that impairment of homologous recombination is not only related to these two genes. Differential expression analysis in sporadic and BRCA-related BCs highlighted two miRNAs (miR-361-5p and miR-151-5p) upregulated in PARP1-overexpressing samples. Recently it was shown that miR-361-5p is upregulated in metastatic BC, but no biological function that could explain such upregulation was highlighted [24]. In our validation study, miR-361-5p upregulation was not confirmed either in BRCA-mutated BCs or in sporadic ones. On the contrary, we observed upregulation of miR-151-5p in PARP1-upregulating BCs also in the validation set. Very few data are available on the role of miR-151-5p in BC. It was proposed as a player in metastasization through the study of its differential expression in primary tumors and the corresponding lymph-node metastases [25]. Furthermore, it was found to have a lower expression in plasma samples with early BC than in healthy-matched controls in a microarray study [26] and in BC serum samples compared to controls through a next generation sequencing approach [27].

In our study, after bioinformatic evaluation of potential target genes of miR-151-5p, we focused on ATM, WEE1 and SMARCA5 genes. ATM [28] and WEE1 [29] are involved in cell cycle arrest, useful for providing time to the cell to repair DNA damage, particularly DSBs. However, these two genes did not prove to be associated with miR-151-5p expression. Only SMARCA5 expression showed an opposite direction compared to that of miR-151-5p in the validation set, and an *in vitro* study confirmed their interaction. Such a result is important because DSB repair acts in the complex tridimensional structure of the chromatin. Indeed, the nucleosome structure physically impairs the detection and the repair of DSBs. The “access-repair-restore” model firstly described the crucial role of chromatin on DNA repair. Compact heterochromatin domains have been shown to have an elevated mutation rate [30], confirming the importance of accessibility to chromatin for DNA repair machinery. SMARCA5 (SWI-SNF-related Matrix-associated Actin-dependent Regulator of Chromatin A5), also known as SNF2H, is one of the two evolutionary conserved ATPase subunits of the ISWI family of ATP-dependent chromatin remodelers. They catalyze the disruption of DNA-histone contacts and control nuclesome assembly and composition acting with histone chaperones [31].

**Figure 5:**
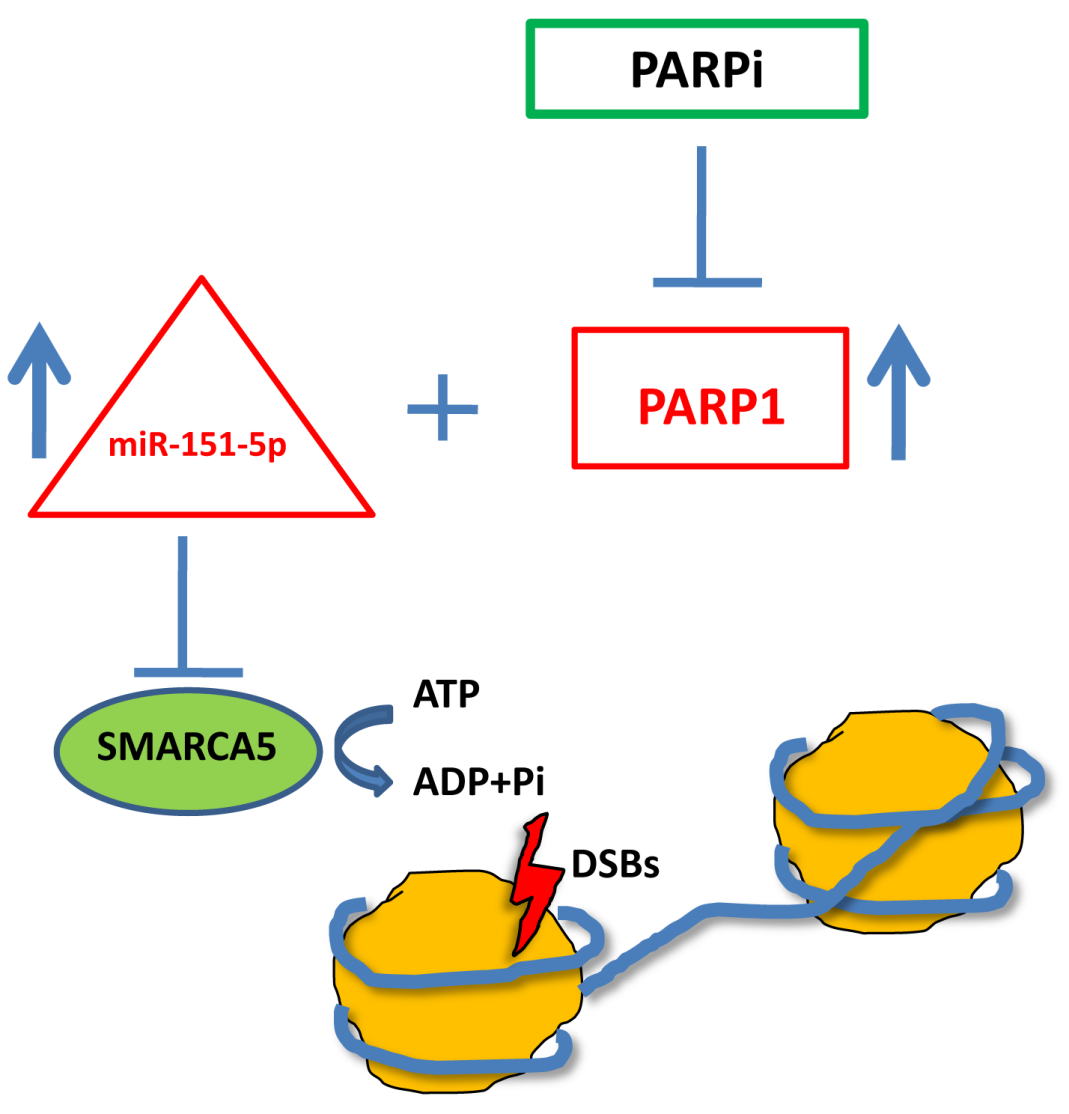
Schematic representation of the proposed model for BRCAness biomarkers

ISWI family members, including SMARCA5, have a role in DDR and, particularly, in DSB repair. SMARCA5 regulates NHEJ and HR through its recruitment with RNF168 at DSB sites in a PARP1-dependent fashion [32]. RNF168-mediated ubiquitylation, which leads to BRCA1 recruitment, is due to SMARCA5 binding to PARylated RNF168. SMARCA5 is recruited to DSB sites also by E3 ubiquitin ligase RNF20 [33] and the deacetylase SIRT6 [34]. So the role of SMARCA5 in DSB repair and in particular in HR is relevant enough to consider the co-upregulation of miR-151-5p and PARP1 as a biomarker for BRCAness patients (Figure 5), that could finally be better directed to PARPi.

## MATERIALS AND METHODS

### Samples

The training set included a cohort of 14 mutated (10 BRCA1-mutated and 4 BRCA2-mutated) and 16 sporadic patients, previously described in [14], enrolled through the Genetic Counseling Program at the IRCCS Istituto Tumori ‘Giovanni Paolo II’ in Bari, Italy. In detail, we considered the same cohort stratified according to the median expression value of PARP1, assessed through RT-PCR. The study was approved by the Ethics Committee of the same Institute as a satellite project of the protocol approved with n. 56/CE of 16/05/2011. Patients signed informed consent giving permission to use their pathological material. The validation set consisting of an independent series of 29 BCs (8 mutated and 21 sporadic cases) was enrolled and classified in the same way as the training set. The normal tissue counterparts of five patients were transformed in the RNA pool used as a calibrator in the real-time PCR experiments. RNA was extracted from fresh frozen cancer specimens containing at least 70% tumour cells and from normal tissues using the RNeasy Plus Mini Kit (Qiagen, Valencia, CA, USA) according to the manufacturer's protocol. Concentrations were estimated with the ND-8000 Spectrophotometer (NanoDrop Technologies, Wilmington, DE, USA).

### MiRNA microarray analysis

500 ng RNA of each sample was labelled using the 3 DNA Array Detection Flash Tag RNA Labelling Kit (Affymetrix, Santa Clara, CA, USA) according to the manufacturer's instructions, and analysed by the Gene Chip miRNA v. 1.0 Array (Affymetrix). This contains 46 228 probes comprising 7815 probe sets and covers 71 organisms including 1100 human miRNAs derived from the Sanger miRBase and miRNA database v11 (15 April 2008, http://microrna.sanger.ac.uk). Firstly, poly (A) tailing was carried out at 37°C for 15 min in a volume of 15 ml reaction mix that contained 1 ml Reaction Buffer, 1.5 ml MgCl2 (25 mM), 1 ml ATP Mix diluted 1:500 and 1 ml PAP enzyme. Subsequently, Flash Tag Ligation was performed at room temperature for 30 min by adding 4 ml of 5 Flash Tag Ligation Mix Biotin and 2 ml T4 DNA Ligase into 15 ml of reaction mix. Next, 2.5 ml of Stop Solution was added to stop the reaction. Each sample was hybridized on the array, washed, stained with the Affymetrix Fluidics Station 450 and scanned with the Affymetrix Gene Chip Scanner 3000 7G using the Command Console software (Affymetrix).

### Array data processing and statistical analysis

Raw data were normalized with the Robust Multiarray Average (RMA) method to remove systematic variations. Briefly, RMA corrects raw data for background using a formula that is based on a normal distribution and uses a linear model to estimate values on a log-scale. The RMA normalization was performed using the ‘Affy’ package of the Bioconductor suite (http://www.bioconductor.org/) for R statistical language (http://cran.r-project.org/). The default settings were used. Normalized values were statistically analyzed with MeV software v.4.8.1 (Dana-Farber Cancer Institute, Boston, MA, USA). Differentially expressed miRNAs were detected through *t*-test, with data considered statistically significant when *p* < 0.01. The microarray data set was deposited in the Array Express database under the accession number E-MTAB-2705.

### Quantitative miRNA RT-PCR analysis

Quantitative RT-PCR analysis of 8 selected miRNAs was performed on an independent series of 8 BRCA-related and 21 sporadic BCs using the TaqMan microRNA Assay (Applied Biosystems, Foster City, CA, USA) on the Applied Biosystems Real-time PCR instrument 7000, in accordance with the manufacturer's instructions (hsa-miR-151-5p assay ID: 002642; hsa-miR-361 assay ID: 000554). Briefly, reverse transcriptase reactions contained: 10 ng of total RNA obtained after RNA isolation, 3 μl RT primers, 1x RT buffer, 100 mM dNTPs, 3.33 U μl^-1^ MultiScribe reverse Transcriptase and 0.25 U μl^-1^ RNase inhibitor. A 20 ml PCR reaction including 3 μl of RT product, 1x TaqMan Universal PCR Master Mix and 1x of the corresponding miRNA assay primers was incubated in 96-well plates at 95°C for 10 min followed by 40 cycles at 95°C for 15 s and 60°C for 1 min. All PCR reactions were performed in triplicate for a technical replicate, including no-template controls, and the mean of the triplicates was used. Relative quantities of each miRNA were calculated using the ΔΔCt method after normalization with endogenous reference RNU48.

### Quantitative RT-PCR analysis of ATM, WEE1, PARP1 and SMARCA5 mRNA levels

The levels of ATM, WEE1, PARP1 and SMARCA5 mRNA were measured using the individual TaqMan RNA Assay (Applied Biosystems) on the Applied Biosystems Real-time PCR instrument 7000 in accordance with the manufacturer's instructions (ATM assay ID: Hs01112307_m1; WEE1 assay ID: Hs01119384_g1; PARP1 assay ID: Hs00242302_m1; SMARCA5 assay ID: Hs00186149_m1). Reverse transcriptase reactions, performed with the High-Capacity cDNA Reverse Transcription Kit (Applied Biosystems), contained 500 ng of total RNA obtained after RNA isolation, 1 × RT Random primers, 1 × RT buffer, 100 mm dNTPs, 1 U *μ*l^−1^ MultiScribe reverse Transcriptase and 1 *μ*l RNase inhibitor. A 20 *μ*l PCR reaction including 2.5 *μ*l of RT product, 1 × TaqMan Universal PCR Master Mix and 1 × of the corresponding RNA assay primers was incubated in 96-well plates at 50 °C for 2 min and at 95 °C for 10 min followed by 40 cycles at 95 °C for 15 s and 60 °C for 1 min. All PCR reactions were performed in triplicate for a technical replicate, including no-template controls, and the mean of the triplicates was used.

Relative quantities of each mRNA were calculated using the ΔΔCt method after normalisation with endogenous reference RNA 18s.

### Dual-luciferase reporter assay and constructs

The MiRWalk database [15c] was used to identify miRNA target genes. The coding sequence of *SMARCA5* was amplified by RT-PCR from HEK293 RNA and cloned into the pmiR-REPORT miRNA Expression Reporter Vector System (TermoFischer). Mutagenesis was carried out to delete *miR-151-5p* binding sites from pmiR-*SMARCA5* by using the QuickChange II kit (Stratagene). All constructs were verified by sequencing. HEK293 cells were transfected by Lipofectamine 2000 (TermoFischer) with indicated constructs (Table 1). After 48 hours, the cells were lysed and assayed for both Firefly and Renilla luciferase activity using the Dual-GLO^®^ Luciferase Assay System (Promega). Firefly luciferase activity was normalized to Renilla luciferase activity for each transfected well. Values are the mean±S.E.M. of three experimental replicates from two to four independent transfections. Significance was determined by a two-tailed paired *t test* for means.

**Table 1:** Sequences of oligos and assays ID used for *in vitro* experiments

Oligos	Sequence
**For cloning**	
Hs_SMARCA F	AGTAGATCTTCAGGCTATGGACC
Hs_SMARCAR	ACTTCTTCTGGAGTTTTGCCTTC
**For mutagenesis**	
Hs_SMARCA_MUT-F	T ATTTC AGGGA AGCTCTTCGTGTT AGTGA A ATGTTC AGGATTTCC AGTTCTTTCCTCC AC
Hs_SM ARCA_MUT-R	GTGGAGGAAAGAACTGGAAATCCTGAACATTTCACTAACACGAAGAGCTTCCCTGAAATA
**For qPCR**	
HS_SMARCA5_RT_F	TCTGTTGCCAGATGTGTTTAATTCA
HS_SMARCA5_RT_R	CCCAAGGCAGTTGTTTGTATCA
**MIMIC and INHIBITOR**	**Commercial code**
mimic hsa-miR-151-5p	SIGMA HMI0244
inhibitor hsa-miR-151 -5p	SIGMA HSTUD0244
mimic control negative	SIGMA HMC0002
inhibitor control negative	SIGMA NCSTUD001

### Quantitative real time reverse transcription-PCR (qPCR)

MCF-7 cells were transfected with *miR-151-5p* mimic or *miR-151-5p* hairpin inhibitor by using Lipofectamine 2000 (TermoFischer). Total RNA was extracted from cells using the RNase mini Kit (TermoFischer) and reverse-transcribed by the Quantitect Transcription kit (Qiagen), according to the manufacturer's instructions. Oligos for qPCR were designed using the Primer express program [35] with default parameters, with *EEF1A1* and *18S* as reference genes. qPCR reactions and calculations were made as reported in [36, 37].

### Computational and statistical analysis

The MiRWalk database [34] was used to identify predicted and validated target genes of the deregulated miRNAs and to show miRNA/target gene interaction. Pathway enrichment analysis was performed through the DAVID 6.7 bioinformatic tool [38]. Data analysis was performed using the GraphPad Prism statistics software package (GraphPad Prism 5.01, San Diego, CA, USA). As the values were not normally distributed, nonparametric tests were used to compare miRNAs and gene expression levels. The Mann-Whitney *U*-test was used to compare the median expression values of miRNAs and genes and two-tailed Fischer's exact test to analyze frequencies. Spearman's test was used to analyze the eventual inverse correlation between miRNA and the target gene. A *P*-value of < 0.05 was considered statistically significant. The Venny2.0 tool was used to draw the Venn diagram (http://bioinfogp.cnb.csic.es/tools/venny/index.html).

## References

[1] Yuan SS, Lee SY, Chen G, Song M, Tomlinson GE, Lee EY (1999). BRCA2 is required for ionizing radiation-induced assembly of Rad51 complex in vivo. Cancer research.

[2] Zhong Q, Chen CF, Li S, Chen Y, Wang CC, Xiao J, Chen PL, Sharp ZD, Lee WH (1999). Association of BRCA1 with the hRad50-hMre11-p95 complex and the DNA damage response. Science.

[3] Byrski T, Gronwald J, Huzarski T, Grzybowska E, Budryk M, Stawicka M, Mierzwa T, Szwiec M, Wisniowski R, Siolek M, Dent R, Lubinski J, Narod S (2010). Pathologic complete response rates in young women with BRCA1-positive breast cancers after neoadjuvant chemotherapy. J Clin Oncol.

[4] Byrski T, Huzarski T, Dent R, Marczyk E, Jasiowka M, Gronwald J, Jakubowicz J, Cybulski C, Wisniowski R, Godlewski D, Lubinski J, Narod SA (2014). Pathologic complete response to neoadjuvant cisplatin in BRCA1-positive breast cancer patients. Breast cancer research and treatment.

[5] Kaelin WG (2005). The concept of synthetic lethality in the context of anticancer therapy. Nature reviews Cancer.

[6] Fong PC, Boss DS, Yap TA, Tutt A, Wu P, Mergui-Roelvink M, Mortimer P, Swaisland H, Lau A, O'Connor MJ, Ashworth A, Carmichael J, Kaye SB, Schellens JH, de Bono JS (2009). Inhibition of poly(ADP-ribose) polymerase in tumors from BRCA mutation carriers. The New England journal of medicine.

[7] Audeh MW, Carmichael J, Penson RT, Friedlander M, Powell B, Bell-McGuinn KM, Scott C, Weitzel JN, Oaknin A, Loman N, Lu K, Schmutzler RK, Matulonis U, Wickens M, Tutt A (2010). Oral poly(ADP-ribose) polymerase inhibitor olaparib in patients with BRCA1 or BRCA2 mutations and recurrent ovarian cancer: a proof-of-concept trial. Lancet.

[8] Tutt A, Robson M, Garber JE, Domchek SM, Audeh MW, Weitzel JN, Friedlander M, Arun B, Loman N, Schmutzler RK, Wardley A, Mitchell G, Earl H, Wickens M, Carmichael J (2010). Oral poly(ADP-ribose) polymerase inhibitor olaparib in patients with BRCA1 or BRCA2 mutations and advanced breast cancer: a proof-of-concept trial. Lancet.

[9] O'shaughnessy J, Osborne C, Pippen JE, Yoffe M, Patt D, Rocha C, Koo IC, Sherman BM, Bradley C (2011). Iniparib plus chemotherapy in metastatic triple-negative breast cancer. The New England journal of medicine.

[10] Guha M (2011). PARP inhibitors stumble in breast cancer. Nature biotechnology.

[11] Smerdon MJ (1991). DNA repair and the role of chromatin structure. Current opinion in cell biology.

[12] Larsen MJ, Thomassen M, Tan Q, Laenkholm AV, Bak M, Sorensen KP, Andersen MK, Kruse TA, Gerdes AM (2014). RNA profiling reveals familial aggregation of molecular subtypes in non-BRCA1/2 breast cancer families. BMC medical genomics.

[13] De Summa S, Pinto R, Sambiasi D, Petriella D, Paradiso V, Paradiso A, Tommasi S (2013). BRCAness: a deeper insight into basal-like breast tumors. Annals of Oncology.

[14] De Summa S, Pinto R, Pilato B, Sambiasi D, Porcelli L, Guida G, Mattioli E, Paradiso A, Merla G, Micale L, De Nittis P, Tommasi S (2014). Expression of base excision repair key factors and miR17 in familial and sporadic breast cancer. Cell Death & Disease.

[15] Dweep H, Sticht C, Pandey P, Gretz N (2011). miRWalk--database: prediction of possible miRNA binding sites by “walking” the genes of three genomes. Journal of biomedical informatics.

[16] Hoeijmakers JH (2009). DNA damage, aging, and cancer. The New England journal of medicine.

[17] Curtin NJ (2012). DNA repair dysregulation from cancer driver to therapeutic target. Nature reviews Cancer.

[18] Farmer H, McCabe N, Lord CJ, Tutt AN, Johnson DA, Richardson TB, Santarosa M, Dillon KJ, Hickson I, Knights C, Martin NM, Jackson SP, Smith GC, Ashworth A (2005). Targeting the DNA repair defect in BRCA mutant cells as a therapeutic strategy. Nature.

[19] Bryant HE, Schultz N, Thomas HD, Parker KM, Flower D, Lopez E, Kyle S, Meuth M, Curtin NJ, Helleday T (2005). Specific killing of BRCA2-deficient tumours with inhibitors of poly(ADP-ribose) polymerase. Nature.

[20] Mendes-Pereira AM, Martin SA, Brough R, McCarthy A, Taylor JR, Kim JS, Waldman T, Lord CJ, Ashworth A (2009). Synthetic lethal targeting of PTEN mutant cells with PARP inhibitors. EMBO molecular medicine.

[21] Pothof J, Verkaik NS, van IW, Wiemer EA, Ta VT, van der Horst GT, Jaspers NG, van Gent DC, Hoeijmakers JH, Persengiev SP (2009). MicroRNA-mediated gene silencing modulates the UV-induced DNA-damage response. The EMBO journal.

[22] Wouters MD, van Gent DC, Hoeijmakers JH, Pothof J (2011). MicroRNAs, the DNA damage response and cancer. Mutation research.

[23] Livraghi L, Garber JE (2015). PARP inhibitors in the management of breast cancer: current data and future prospects. BMC medicine.

[24] Sun EH, Zhou Q, Liu KS, Wei W, Wang CM, Liu XF, Lu C, Ma DY (2014). Screening miRNAs related to different subtypes of breast cancer with miRNAs microarray. European review for medical and pharmacological sciences.

[25] Krell J, Frampton AE, Jacob J, Pellegrino L, Roca-Alonso L, Zeloof D, Alifrangis C, Lewis JS, Jiao LR, Stebbing J, Castellano L (2012). The clinico-pathologic role of microRNAs miR-9 and miR-151-5p in breast cancer metastasis. Molecular diagnosis & therapy.

[26] Zhao H, Shen J, Medico L, Wang D, Ambrosone CB, Liu S (2010). A pilot study of circulating miRNAs as potential biomarkers of early stage breast cancer. PloS one.

[27] Wu Q, Wang C, Lu Z, Guo L, Ge Q (2012). Analysis of serum genome-wide microRNAs for breast cancer detection. Clinica chimica acta; international journal of clinical chemistry.

[28] Lavin MF (2008). Ataxia-telangiectasia: from a rare disorder to a paradigm for cell signalling and cancer. Nature reviews Molecular cell biology.

[29] Russell P, Nurse P (1987). Negative regulation of mitosis by wee1+, a gene encoding a protein kinase homolog. Cell.

[30] Schuster-Bockler B, Lehner B (2012). Chromatin organization is a major influence on regional mutation rates in human cancer cells. Nature.

[31] Aydin OZ, Vermeulen W, Lans H (2014). ISWI chromatin remodeling complexes in the DNA damage response. Cell cycle.

[32] Smeenk G, Wiegant WW, Marteijn JA, Luijsterburg MS, Sroczynski N, Costelloe T, Romeijn RJ, Pastink A, Mailand N, Vermeulen W, van Attikum H (2013). Poly(ADP-ribosyl)ation links the chromatin remodeler SMARCA5/SNF2H to RNF168-dependent DNA damage signaling. Journal of cell science.

[33] Nakamura K, Kato A, Kobayashi J, Yanagihara H, Sakamoto S, Oliveira DV, Shimada M, Tauchi H, Suzuki H, Tashiro S, Zou L, Komatsu K (2011). Regulation of homologous recombination by RNF20-dependent H2B ubiquitination. Molecular cell.

[34] Toiber D, Erdel F, Bouazoune K, Silberman DM, Zhong L, Mulligan P, Sebastian C, Cosentino C, Martinez-Pastor B, Giacosa S, D'Urso A, Naar AM, Kingston R, Rippe K, Mostoslavsky R (2013). SIRT6 recruits SNF2H to DNA break sites, preventing genomic instability through chromatin remodeling. Molecular cell.

[35] Rozen S, Skaletsky H (2000). Primer3 on the WWW for general users and for biologist programmers. Methods in molecular biology.

[36] Micale L, Fusco C, Fontana A, Barbano R, Augello B, De Nittis P, Copetti M, Pellico MT, Mandriani B, Cocciadiferro D, Parrella P, Fazio VM, Dimitri LM, D'Angelo V, Novielli C, Larizza L (2015). TRIM8 downregulation in glioma affects cell proliferation and it is associated with patients survival. BMC cancer.

[37] Ferrero GB, Howald C, Micale L, Biamino E, Augello B, Fusco C, Turturo MG, Forzano S, Reymond A, Merla G (2010). An atypical 7q11. 23 deletion in a normal IQ Williams-Beuren syndrome patient. Eur J Hum Genet.

[38] Huang da W, Sherman BT, Lempicki RA (2009). Systematic and integrative analysis of large gene lists using DAVID bioinformatics resources. Nature protocols.

